# Lysosome–autophagy networks in lung development, maturation, and pediatric lung disease

**DOI:** 10.3389/fped.2026.1967172

**Published:** 2026-10-19

**Authors:** Yajie Song, Dingning Zhang, Jiahui Bao, Guiyang Jiang, Dan Zhang

**Affiliations:** 1 Department of Pediatrics, Shengjing Hospital of China Medical University, Shenyang, Liaoning, China; 2 Department of Pathology, The First Hospital and The College of Basic Medical Sciences of China Medical University, Shenyang, Liaoning, China

**Keywords:** alveolar type II cells, autophagy, lung development, lysosome, pediatric lung disease, pulmonary surfactant

## Abstract

Lysosomes are increasingly recognized as dynamic regulatory organelles that integrate degradation, nutrient sensing, lipid metabolism, membrane trafficking, and organelle quality control beyond their classical degradative functions. These activities are particularly important during lung development, when epithelial differentiation, alveolarization, lung maturation, surfactant homeostasis, and postnatal adaptation occur within a narrow developmental window. This review summarizes current evidence linking lysosome–autophagy networks to lung maturation, with emphasis on epithelial autophagy, alveolar type II (AT-II) cell function, lamellar-body biology, mitochondrial quality control, and alveolar macrophage regulation. We discuss how lysosomal lipid handling, autophagic flux, and mechanistic target of rapamycin (mTOR)–transcription factor EB (TFEB) signaling contribute to developmental homeostasis while distinguishing direct pulmonary evidence from mechanistic hypotheses derived from other systems. We further examine lysosomal and autophagic alterations in pediatric lung diseases, including bronchopulmonary dysplasia, cystic fibrosis, and viral respiratory infection, highlighting disease-specific upstream stressors and intracellular defects. Static autophagy markers cannot define functional flux, and conventional lysosomes must be distinguished from specialized lysosome-related organelles such as lamellar bodies. Finally, we discuss therapeutic opportunities and challenges, emphasizing cell-specific targeting, restoration of defined organelle defects, developmental safety, and rigorous functional validation. A developmentally informed understanding of lysosome–autophagy networks may provide new mechanistic frameworks for pediatric lung disease intervention.

## 1 Background and overview of lysosome–autophagy networks in lung biology

The overall organization of lysosome–autophagy networks in lung development and pediatric lung disease is summarized in Figure 1.

**Figure 1:**
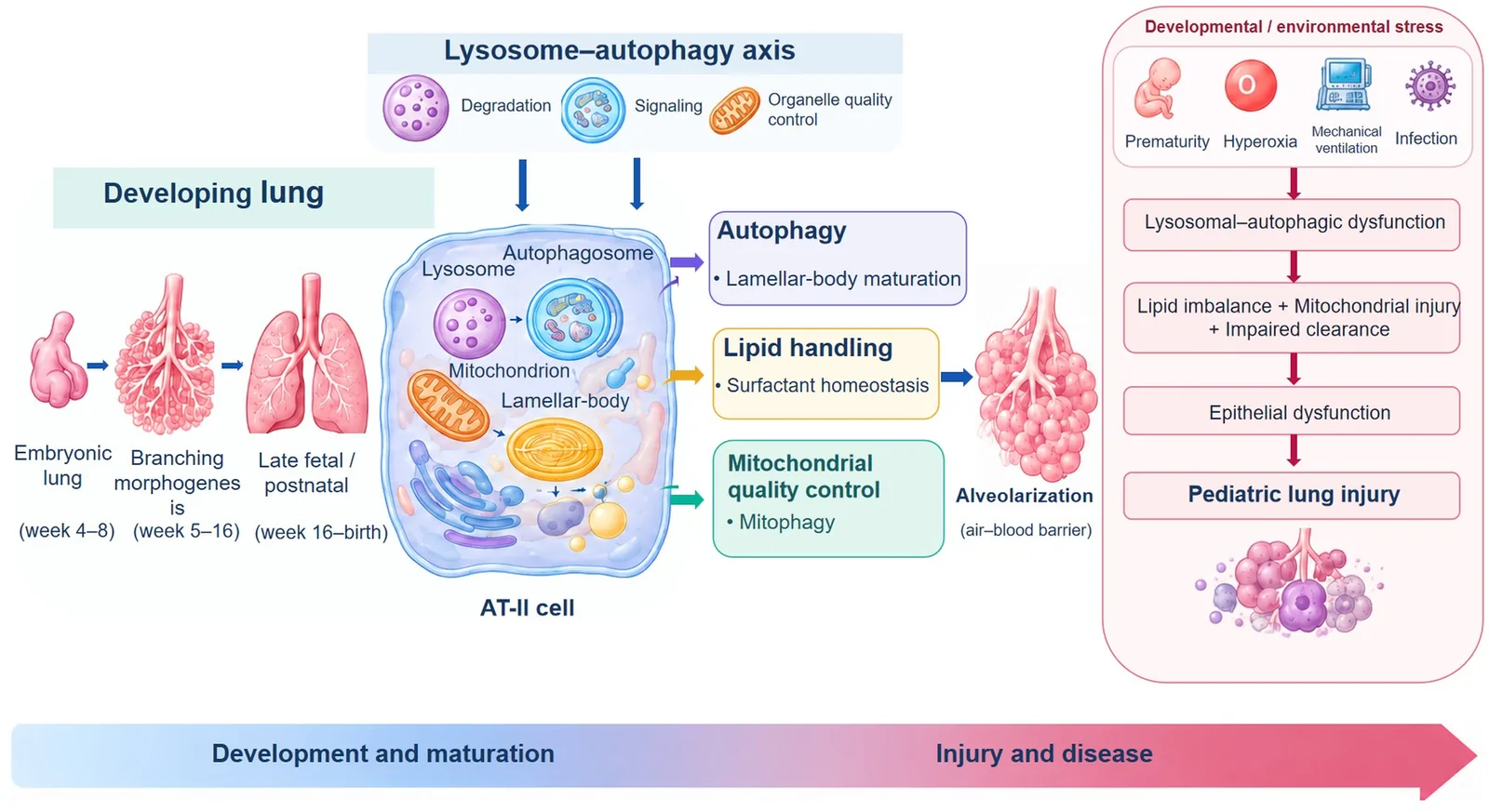
Integrated lysosome–autophagy networks in lung development and pediatric lung disease.

### 1.1 Lysosomes as dynamic regulatory organelles beyond degradation

Lysosomes are acidic, single-membrane organelles containing a broad repertoire of hydrolases that degrade proteins, lipids, nucleic acids, and other cellular cargo. Their function depends on coordinated luminal acidification, lysosome-associated membrane proteins 1 and 2 (LAMP1 and LAMP2), proton-pumping vacuolar-type H⁺-ATPase (V-ATPase) complexes, and trafficking pathways that deliver endocytic and autophagic cargo to the degradative compartment (1–5). Beyond degradation, lysosomes regulate nutrient sensing, metabolite recycling, organelle quality control, membrane trafficking, and selected inflammatory responses. They therefore act as dynamic regulatory platforms whose effects depend on cellular state and intracellular compartment.

Macroautophagy is the best-characterized lysosome-dependent recycling pathway. Cytoplasmic cargo is enclosed within double-membrane autophagosomes, which subsequently fuse with lysosomes to form autolysosomes in which cargo is degraded and recycled. Microautophagy and chaperone-mediated autophagy provide additional routes for lysosomal cargo delivery. In the developing lung, these pathways are relevant because epithelial cells must simultaneously maintain organelle quality, adapt to changing nutrient and oxygen availability, and acquire specialized secretory functions. Mitophagy is a selective form of autophagy that delivers mitochondria to lysosomes for degradation and contributes to mitochondrial quality control. Its role in normal lung maturation must be distinguished from context-dependent responses observed in pulmonary injury models, as discussed in Section 2.1.

A key methodological consideration is the interpretation of autophagy-related markers. Increased levels of lipidated microtubule-associated protein 1 light chain 3 (LC3-II) or p62/sequestosome 1 (p62/SQSTM1), or increased autophagosome abundance, may result either from increased autophagosome formation or from impaired downstream degradation. Accordingly, static measurements cannot by themselves establish productive autophagic activity; conclusions about pathway activation or inhibition should be supported, where possible, by dynamic flux measurements or complementary turnover assays (6). This distinction is particularly important when lysosomal dysfunction is proposed as a mechanism of pediatric lung disease.

### 1.2 The lysosome–autophag*y* axis as a core hub in lung physiology and development

The developing lung undergoes rapid structural and functional transitions, including epithelial differentiation, alveolarization, pulmonary microvascular maturation, establishment of surfactant competence, and adaptation to air breathing after birth. These processes place high demands on intracellular membrane trafficking and organelle quality control. Conditional pulmonary studies directly implicate epithelial autophagy in lung branching and morphogenesis (7). In AT-II cell models, disruption of autophagy-related pathways impairs lamellar-body maturation and surfactant homeostasis (8, 9). The mechanisms connecting epithelial autophagy to vascular maturation require further cell-specific study.

The developmental context also determines how lysosomal stress should be interpreted. Premature infants may enter extrauterine life before alveolar and microvascular maturation is complete, making the immature lung particularly sensitive to hyperoxia, mechanical stress, inflammation, and infection. Experimental models show that these insults can alter autophagy, mitochondrial quality control, and lysosomal responses, but the direction of change is not uniform. Thus, lysosomal pathways should not be viewed as intrinsically protective or pathogenic; their effects depend on cell type, developmental stage, cargo, and the balance between formation and clearance.

Lysosome–autophagy networks integrate degradation, nutrient sensing, organelle quality control, membrane trafficking, and inflammatory signaling in developing lungs. Rather than representing a single autonomous regulatory pathway, these networks interact with cell-specific developmental programs and disease-associated stresses. Their effects depend on cellular context, intracellular compartment, developmental stage, and the balance between cargo delivery and degradation.

## 2 Core components and functional roles of lysosome–autophagy networks in lung development

### 2.1 Epithelial organelle homeostasis, lipid metabolism, and surfactant biology

Lysosomal lipid handling provides a direct example of how lysosomal function can influence pulmonary epithelial phenotype. Lysosomal acid lipase (LAL), encoded by LIPA, hydrolyzes cholesteryl esters and triglycerides within lysosomes and thereby contributes to intracellular neutral-lipid homeostasis. In the lung, respiratory epithelial overexpression of human LAL disrupts surfactant composition, reduces neutral lipid content in bronchoalveolar lavage fluid, depletes lamellar bodies in AT-II cells, and is associated with alveolar simplification and airspace enlargement (10). These findings indicate that excessive lysosomal lipid catabolism can disturb the lipid environment required for surfactant homeostasis and alveolar structure.

These data do not establish that lysosomal degradation is the principal source of substrate for *de novo* surfactant synthesis. Rather, they demonstrate that the magnitude and compartmentalization of lysosomal lipid catabolism can influence the cellular lipid pool, lamellar-body biology, and alveolar architecture. In this sense, lysosomes should be considered one component of a distributed lipid-trafficking network that includes the endoplasmic reticulum, Golgi apparatus, lipid transporters, lamellar bodies, and extracellular surfactant recycling.

The sensitivity of AT-II cells to lysosomal lipid imbalance is also illustrated by respiratory syncytial virus (RSV) infection, in which impaired LAL activity promotes lysosomal cholesterol accumulation, activation of the sterol regulatory element-binding protein 2 (SREBP2)–low-density lipoprotein receptor (LDLR) axis, defective acidification, and impaired autophagic flux (11). This disease-model finding complements the LAL-overexpression phenotype but does not establish a mechanism of normal lung development; the infection-specific pathway is discussed in Section 3.3.

Pulmonary surfactant is synthesized through coordinated endoplasmic reticulum (ER), Golgi, and lipid metabolic pathways and is packaged, stored, and secreted by AT-II cells. Lamellar bodies (LBs) are specialized lysosome-related organelles (LROs) that serve as major intracellular sites for surfactant storage and secretion and should therefore be distinguished from conventional degradative lysosomes (12, 13). Their specialized composition and trafficking machinery allow AT-II cells to maintain surfactant homeostasis while responding rapidly to changes in alveolar mechanical forces.

LB function depends on specialized membrane transport systems and an acidic lumen, with a reported pH of approximately 5.0–5.5 (12, 13). V-ATPase-dependent proton transport contributes to LB function (14), but this study should not be interpreted as an independent measurement of that precise pH range. Acidic conditions support surfactant-protein processing, while coordinated lipid transport and packaging establish the phospholipid-rich material needed to reduce alveolar surface tension (12–14). ER membrane protein complex subunit 3 (EMC3) supports ATP-binding cassette subfamily A member 3 (ABCA3) biogenesis and surfactant protein and lipid homeostasis, whereas transmembrane proteins 63A and 63B (TMEM63A and TMEM63B) participate in mechanically stimulated LB secretion during lung inflation (15, 16). These processes are summarized in Figure 2.

**Figure 2:**
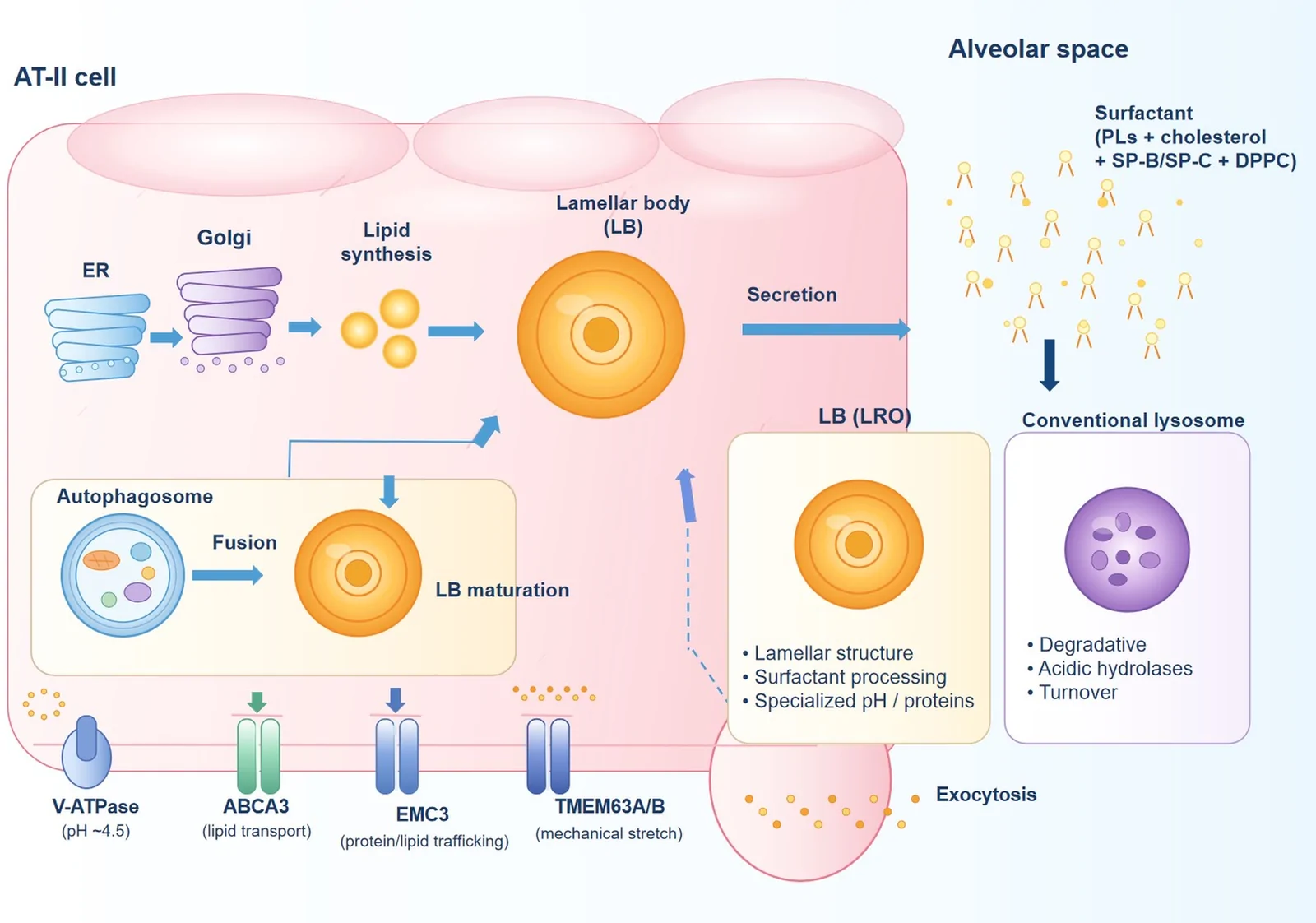
Lamellar body biogenesis and pulmonary surfactant homeostasis in alveolar type II cells.

Lamellar bodies (LBs) are specialized lysosome-related organelles (LROs) in alveolar type II (AT-II) cells that coordinate surfactant storage, processing, and secretion. Endoplasmic reticulum (ER)- and Golgi-dependent lipid synthesis provides substrates for LB formation, while autophagy contributes to LB maturation through fusion-dependent membrane remodeling. LBs have an acidic lumen (approximately pH 5.0–5.5) and are distinct from conventional degradative lysosomes (13).

LB function requires coordinated regulation of acidification, lipid transport, and membrane trafficking. V-ATPase-mediated proton transport, ABCA3-dependent lipid transport, EMC3-associated surfactant protein/lipid homeostasis, and mechanosensitive TMEM63A/B-mediated secretion collectively maintain surfactant homeostasis. Mature LBs release phospholipids, including dipalmitoylphosphatidylcholine (DPPC), together with cholesterol and surfactant proteins into the alveolar space to reduce surface tension and support pulmonary adaptation.

Genetic and cell-biological studies further connect autophagy to LB biology. Autophagosomes can interact with developing LBs, and loss of core autophagy components such as FAK family-interacting protein of 200 kDa (FIP200) or autophagy-related protein 7 (ATG7) disrupts LB maturation and pulmonary surfactant homeostasis (8, 9). Together with conditional evidence that epithelial autophagy is required for lung morphogenesis (7), these findings support roles in organelle maturation and epithelial secretory competence. Autophagy should therefore be distinguished from the upstream synthesis of surfactant lipids and proteins.

Evidence from a multiwalled carbon nanotube injury model links lysosomal damage, altered pH, impaired autolysosome formation, and p62 accumulation to defective LB maturation and reduced secretion of DPPC and surfactant protein C (SP-C) (17). Because this is an injury model, it supports a possible failure mechanism rather than proving the same sequence during normal lung development.

These epithelial functions become particularly important around birth, when the lung adapts to air breathing, mechanical stretch, and changing nutrient and oxygen availability. Whole-body neonatal studies show that autophagy is rapidly induced after birth and is required for survival during the early nutrient-deprivation period (18). These systemic findings provide context for postnatal adaptation but do not independently establish a lung-cell-specific mechanism.

Within this broader organelle-homeostasis framework, mitophagy specifically concerns lysosomal clearance of mitochondria. Its contribution to normal epithelial maturation remains less directly established than the role of general autophagy in LB biology. In hyperoxia-exposed AT-II cells, autophagy has been studied in relation to intracellular SP-C accumulation (19), but this does not independently demonstrate a mitophagy mechanism. A separate hyperoxia-induced acute lung injury study found that growth arrest and DNA damage-inducible gene 7 (gadd7)–mitofusin 1 (MFN1)-associated mitophagy promoted AT-II cell apoptosis (20). Thus, injury-model findings do not establish a uniformly protective mitophagy program during normal neonatal lung maturation; direct cell-specific developmental studies are needed.

Epithelial lipid handling, LB maturation, autophagic turnover, and mitochondrial quality control form an interconnected framework for pulmonary adaptation around birth, with different levels of direct developmental evidence. Disease-specific disruptions are discussed in Section 3, and the mechanisms and evidence boundaries are summarized in Table 1.

**Table 1:** Molecular mechanisms and evidence boundaries of lysosome–autophagy regulation in lung development and maturation.

Mechanism	Pulmonary relevance	Representative evidence/models	Current evidence limitations
Epithelial autophagy during lung morphogenesis	Epithelial autophagy contributes to pulmonary branching and morphogenesis; its precise cell-specific links to vascular maturation remain to be defined.	Conditional pulmonary epithelial autophagy model demonstrating impaired lung morphogenesis (7)	The precise cell-type-specific mechanisms linking autophagy to developmental patterning remain incompletely defined.
Autophagy–lamellar body interaction in AT-II cells	Autophagy supports lamellar-body maturation, surfactant processing, and maintenance of AT-II cell secretory function.	FIP200- and ATG7-related AT-II cell models demonstrating impaired lamellar-body maturation and surfactant homeostasis (8, 9)	Autophagy regulates organelle maturation and quality control but is not the site of *de novo* surfactant lipid synthesis.
Lysosomal lipid handling and surfactant homeostasis	Lysosomal lipid metabolism influences intracellular lipid balance, lamellar-body abundance, surfactant composition, and alveolar structure.	Respiratory epithelial LAL overexpression models showing altered surfactant homeostasis and alveolar abnormalities (10)	The effects of lysosomal lipid metabolism depend on lipid flux, cellular context, and developmental stage.
Mitophagy and mitochondrial quality control	Autophagy and mitophagy respond to hyperoxic AT-II cell stress, but their effects on survival can differ by pathway and model.	AT-II cell SP-C accumulation under hyperoxia (19); gadd7–MFN1-associated mitophagy promoting AT-II cell apoptosis in hyperoxic acute lung injury (20)	A protective developmental role for mitophagy has not been established; hyperoxic injury studies report context-dependent effects.
mTOR–TFEB signaling and lysosomal adaptation	mTOR–TFEB signaling links nutrient sensing with lysosomal biogenesis, autophagy regulation, and cellular stress adaptation.	General mTOR–TFEB mechanistic studies and experimental BPD models (31–36)	Direct evidence for an AT-II cell–specific TFEB developmental program remains limited.

AT-II, alveolar type II; BPD, bronchopulmonary dysplasia; LAL, lysosomal acid lipase. Evidence limitations indicate areas where mechanisms remain incompletely established in developing lung models and should not be interpreted as established causal pathways.

Evidence from other tissues suggests that lysosomal degradation can influence lineage-regulating proteins. This comparative observation is hypothesis-generating: a corresponding lysosome-dependent AT-II cell lineage mechanism has not been demonstrated in the developing lung.

### 2.2 Alveolar macrophage development and tissue remodeling

Direct evidence from developing lung models establishes that macrophage biology intersects with postnatal structural maturation, although it does not yet identify a lysosomal cause. Fetal monocytes acquire an alveolar macrophage phenotype around birth through granulocyte–macrophage colony-stimulating factor (GM-CSF)-dependent maturation (21). In a mouse model with epithelial *β*1-integrin deletion, macrophage depletion reduced persistent inflammation and rescued defective secondary septation (22). In neonatal hyperoxia, depletion experiments implicated the colony-stimulating factor 1 receptor (Csf1r)-expressing monocyte/macrophage lineage in arrested alveolarization (23). These studies link macrophage state to alveolar structure under developmental perturbation, but none establishes that macrophage lysosomal activity independently directs normal alveolarization.

Evidence from nondevelopmental particle and inflammatory models offers testable mechanisms rather than a demonstrated neonatal lung pathway. Phagolysosomal damage and endosomal receptor signaling can change macrophage inflammatory responses (24, 25). Acute lung inflammation has also been examined in relation to alveolar macrophage death (26), while separate particle or infection models link lysosomal injury to mitochondrial and transcriptional responses (27, 28). Such changes might affect the environment for epithelial and vascular maturation, but lysosome-specific manipulations in neonatal alveolar macrophages have not yet been linked directly to structural lung outcomes.

The next test is to perturb a defined lysosomal step specifically in neonatal alveolar macrophages, verify its effect on clearance or signaling, and then measure secondary septation, epithelial differentiation, and vascular development. This would distinguish a macrophage lysosomal contribution to lung maturation from a broader effect of macrophage depletion or inflammation.

### 2.3 mTOR–TFEB signaling and transcriptional adaptations

mTOR signaling provides an important interface between nutrient availability, growth, autophagy, and lysosomal function. During lung morphogenesis, mTOR contributes to airway branching, epithelial differentiation, and coordination between epithelial and vascular development (29, 30). These developmental effects are well supported, but they should not automatically be interpreted as consequences of lysosomal positioning. The lysosome–mTOR relationship is best viewed as one component of a broader developmental signaling network.

Amino acids recruit mTOR complex 1 (mTORC1) to the lysosomal surface through Ragulator–Rag, connecting nutrient sensing to autophagy and TFEB-dependent lysosomal gene expression (31–34). Lysosomal positioning can also affect autophagosome–lysosome encounters. This cellular mechanism offers a developmental framework, but its AT-II cell–specific contribution to morphogenesis remains to be established.

Direct evidence from the developing lung concerns epithelial autophagy and lamellar-body maturation (7–9). Separate neonatal hyperoxia studies report improved structural outcomes after autophagy induction (35) or TFEB-linked intervention (36); these injury-model findings do not by themselves establish a TFEB-dependent program in normal lung development.

Stage- and cell-specific perturbation with rescue is needed to determine whether TFEB affects AT-II differentiation directly or supports adaptation after developmental stress.

The key developmental question is whether nutrient-responsive lysosomal capacity preserves epithelial and vascular maturation; therapeutic applications are considered in Section 4.

## 3 Lysosomal homeostasis imbalance in pediatric lung diseases

Bronchopulmonary dysplasia (BPD), cystic fibrosis (CF), and viral respiratory injury involve distinct upstream stressors and endolysosomal or autophagic alterations that may converge on impaired clearance, disturbed surfactant homeostasis, mitochondrial injury, and epithelial dysfunction. The following subsections connect each disease driver with the affected intracellular process and observed phenotype; these convergent outcomes do not establish a shared primary lysosomal lesion. Figure 3 summarizes the disease-specific alterations alongside the normal developmental context.

**Figure 3:**
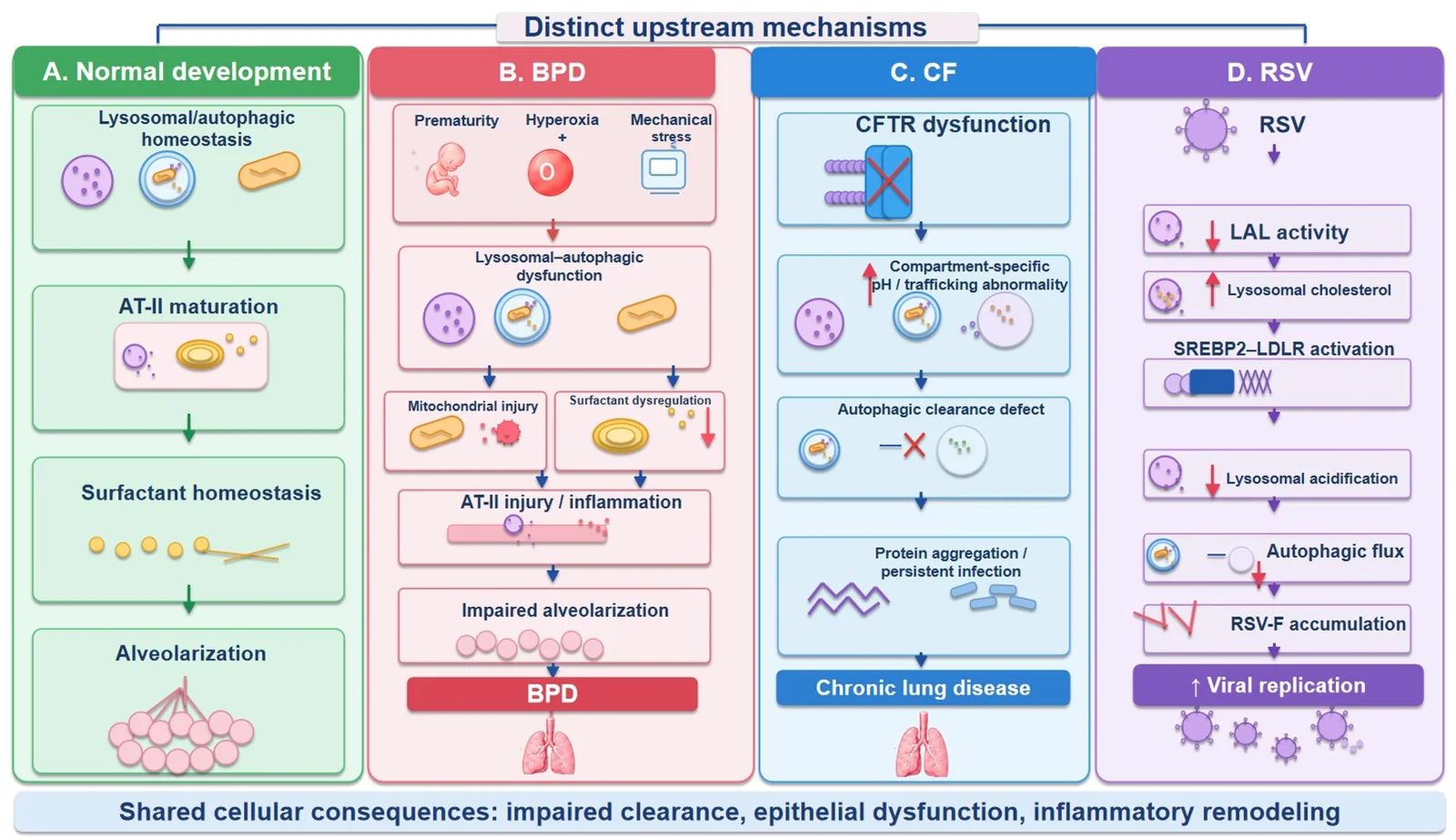
Disease-specific lysosome–autophagy alterations in pediatric lung disorders (section 3).

Lysosome–autophagy pathways participate in pulmonary epithelial homeostasis during normal development, but disease-associated alterations arise through distinct upstream mechanisms. BPD, CF, and viral respiratory infections should therefore not be interpreted as sharing a unified lysosomal defect. Instead, each condition involves specific perturbations of lysosomal trafficking, autophagic clearance, lipid metabolism, or inflammatory signaling that converge on epithelial dysfunction.

### 3.1 Bronchopulmonary dysplasia: developmental disruption of lysosomal adaptation

Figure 3 summarizes the lysosomal and autophagic alterations reported in experimental BPD models.

BPD is a multifactorial disorder of the developing lung in which prematurity, oxygen exposure, mechanical ventilation, inflammation, nutritional factors, and genetic susceptibility interact to disrupt alveolarization and pulmonary vascular development (37, 38). Lysosomal and autophagic pathways are increasingly implicated in experimental models, but no single lysosomal abnormality explains the clinical syndrome. The relevant question is therefore not whether autophagy is globally increased or decreased, but which cell, compartment, and functional step is altered.

Experimental BPD studies differ in developmental stage, injury intensity, experimental model, cell population analyzed, and whether they measure autophagy initiation or completed lysosomal degradation. These distinctions are essential when comparing the findings below; static autophagy markers alone cannot establish the direction of autophagic flux (6).

Experimental BPD studies report heterogeneous phenotypes, including impaired autophagic activity, altered AMP-activated protein kinase (AMPK) signaling, defective clearance, stress-associated autophagy, altered mitophagy, and inflammatory remodeling (19, 35, 36, 39–42). In murine and baboon models, impaired autophagic activity has been associated with BPD pathogenesis, while autophagy-inducing interventions can improve selected measures of alveolarization in hyperoxia-exposed neonatal animals (35, 39). These divergent observations require dynamic flux measurements and compartment-specific analyses. They do not establish that nonspecific activation of the pathway is universally beneficial.

In AT-II cells, hyperoxia-induced intracellular SP-C accumulation has been linked to altered autophagy (19). That study does not independently establish a protective mitophagy response or directly quantify mitochondrial injury. A separate hyperoxia-induced acute lung injury model found that gadd7–MFN1-associated mitophagy promoted AT-II cell apoptosis (20). Other experimental BPD studies implicate macrophage AMPK-dependent autophagy, methyltransferase-like 3 (METTL3)-related suppression of autophagy-related protein 8 (ATG8)-mediated autophagy, and TFEB-linked mitochondrial clearance (36, 40, 41). Hyperoxia-induced transmembrane protein 106B (TMEM106B) has also been reported to impair Unc-51-like autophagy-activating kinase 1 (ULK1)-mediated autophagy through Toll-like receptor 3 (TLR3)/c-Jun N-terminal kinase (JNK) signaling (42). The direction of effect therefore depends on cell type, pathway, and injury model; most evidence remains preclinical.

The emerging therapeutic evidence is therefore best interpreted as pathway matching rather than generic autophagy stimulation. For example, itaconic acid regulation of TFEB-mediated autophagic flux has been reported to alleviate hyperoxia-induced BPD in experimental models (36). Such findings justify further investigation of TFEB and related pathways, but therapeutic direction should be determined by direct measurement of the underlying flux or lysosomal defect rather than by diagnosis alone.

The structural phenotype of BPD—simplified alveoli and abnormal pulmonary vascular development—also emphasizes the developmental consequences of intracellular dysfunction. However, clinical manifestations should not be attributed directly to lysosomal dysfunction without validation of a measurable lysosomal disease endotype. This distinction is essential for translating experimental lysosome biology into neonatal medicine.

### 3.2 Cystic fibrosis: intracellular trafficking and autophagic dysfunction

Figure 3 summarizes trafficking and autophagic alterations associated with dysfunction of the cystic fibrosis transmembrane conductance regulator (CFTR).

CF provides a useful example of why lysosomal function must be interpreted at the level of the specific intracellular compartment. CFTR mutations disrupt epithelial ion transport, mucus hydration, mucociliary clearance, and host defense. Early studies reported abnormal intracellular organelle acidification, whereas subsequent work identified hyperacidification of selected endocytic compartments and direct measurements showing that lysosomal pH may remain unchanged after CFTR loss or inhibition (43–45). Thus, lysosomal, endosomal, Golgi, and airway surface liquid (ASL) pH should not be treated as interchangeable variables.

CFTR dysfunction can also alter autophagy and bacterial clearance independently of bulk lysosomal pH. Defective CFTR has been associated with reactive oxygen species (ROS)-mediated inhibition of autophagy and aggresome formation, while syntaxin-17-dependent pathways can influence autophagosomal clearance under stress (46, 47). These observations suggest that therapeutic interpretation should focus on the specific trafficking or clearance defect rather than assuming that global lysosomal acidification is abnormal.

The CF example is therefore valuable for pediatric lung biology not because it establishes a universal lysosomal pH defect, but because it demonstrates the importance of compartment-resolved measurements. Therapeutic strategies that alter lysosomal or endosomal acidification should be evaluated against the relevant intracellular phenotype and balanced against potential effects on host defense and epithelial homeostasis.

### 3.3 Viral respiratory infection and lysosomal host–pathogen interaction

Figure 3 summarizes virus-associated changes in lysosomal activity and autophagic flux relevant to host–pathogen interactions.

Respiratory viruses can remodel endolysosomal trafficking, lipid metabolism, and autophagy, but the direction and consequence of these changes depend on the virus, cell type, and stage of infection. RSV provides a particularly clear example. RSV infection has been reported to reduce LAL activity, impair cholesterol transport from lysosomes to the ER, activate the SREBP2–LDLR axis, and promote cholesterol accumulation within lysosomes. Cholesterol enrichment is associated with impaired lysosomal acidification and blocked autophagic flux, resulting in retention of the RSV fusion protein and enhanced viral replication (11).

Other viruses exploit endolysosomal biology differently. Betacoronaviruses can use lysosomal trafficking for non-lytic egress, a process associated with lysosomal deacidification and impaired degradation (48). Severe acute respiratory syndrome coronavirus 2 (SARS-CoV-2) open reading frame 3a (ORF3a) has been reported to inhibit autophagosome–lysosome fusion (49). These distinct steps should not be generalized across pediatric viral pneumonias or conflated with virus entry.

In developing airways, lysosomal stress may additionally interact with epithelial barrier injury and inflammatory signaling. However, much mechanistic evidence still derives from immortalized cells or adult models. Age-appropriate airway and lung models, together with dynamic measurements of autophagic flux and compartment-specific lysosomal function, are needed to establish how these pathways influence pediatric disease severity.

## 4 Therapeutic opportunities and translational challenges

### 4.1 Restoring lysosome–autophagy homeostasis

Intervention should begin with a defined defect in autophagosome formation, fusion, lysosomal acidification, or cargo degradation. Figure 4 maps the mTORC1–TFEB pathway onto potential points of intervention; Section 2.3 describes its developmental signaling role. A change in pathway markers alone is insufficient to establish that lysosomal function has been restored.

**Figure 4:**
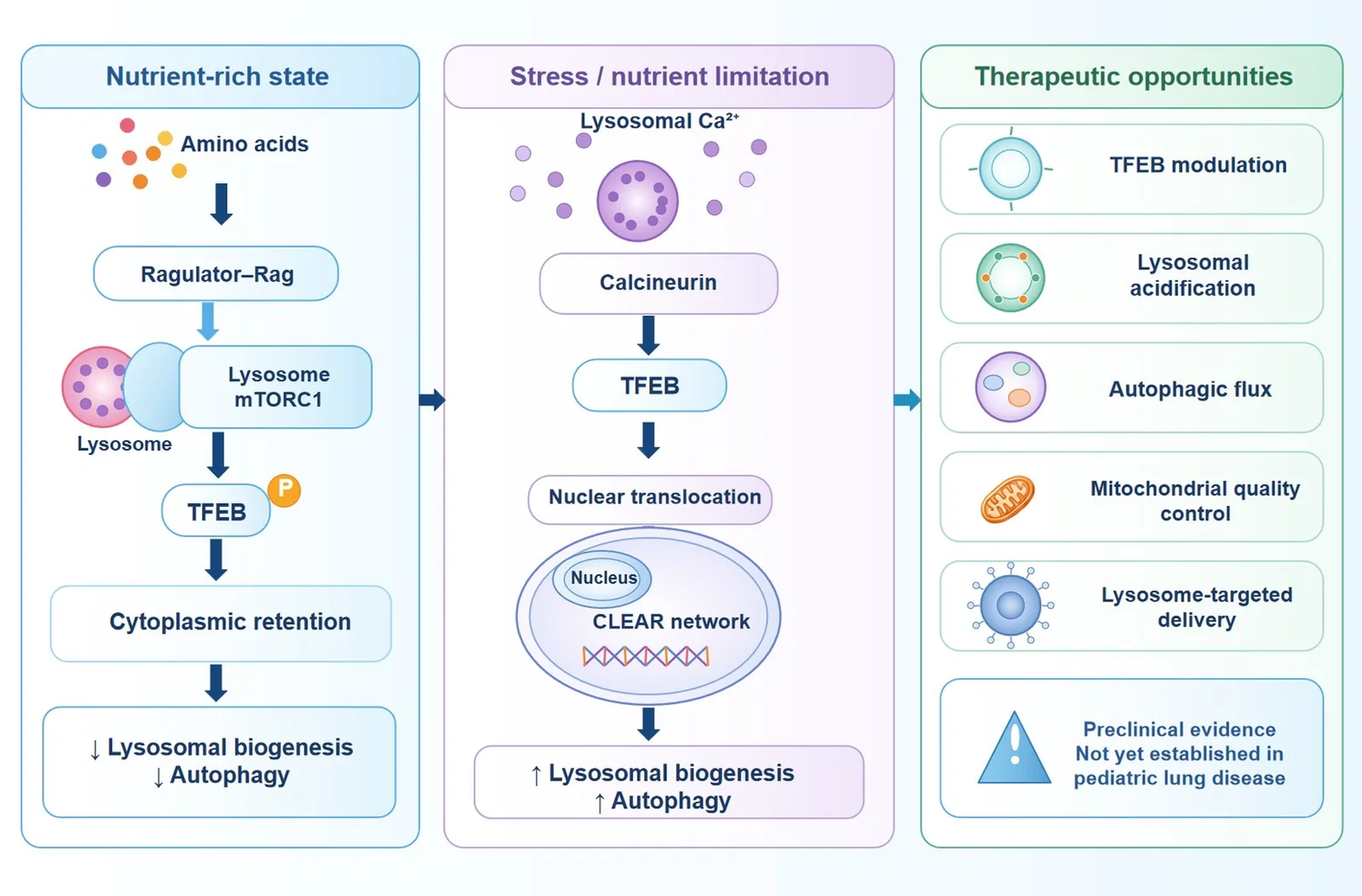
mTORC1–TFEB signaling links lysosomal homeostasis to therapeutic opportunities in pediatric lung disease.

Lysosome-targeted intervention is most plausible when a specific lysosomal or autophagic defect has been demonstrated. The clinical success of lysosome-directed therapies in selected lysosomal storage disorders establishes the tractability of the organelle as a therapeutic target, but these successes cannot be directly extrapolated to BPD, CF, or other pediatric lung diseases (50). In the developing lung, pathway manipulation must preserve normal organelle turnover, host defense, epithelial differentiation, and surfactant function.

Experimental BPD studies provide an initial proof of principle. Autophagy-inducing interventions and TFEB-linked modulation have improved selected outcomes in neonatal hyperoxia models (35, 36). The translational priority is therefore not broad activation of the lysosome–autophag*y* axis, but identification of the defective step and restoration of appropriate flux. This distinction is particularly important because excessive or misdirected lysosomal activation could alter developmental signaling or compromise cellular viability.

### 4.2 Targeting mTOR–TFEB and autophagy-related pathways

TFEB-directed treatment is a candidate approach when impaired lysosomal capacity or flux has been demonstrated in the relevant cell type. Neonatal hyperoxia studies report improved outcomes after TFEB-linked modulation (36), but they do not establish efficacy in human BPD or justify universal TFEB activation. Candidate agents require evidence of restored flux and improved lung structure, with parallel assessment of epithelial differentiation, surfactant function, and immune defense.

Lysosomal acidification is a potential therapeutic target, but its manipulation requires compartment-specific measurements. Studies in CF have reported different pH phenotypes in endocytic and lysosomal compartments (43–45). Separately, V-ATPase contributes to acidification of the ASL in pig small airway epithelial cells (51); this extracellular measurement does not establish the pH of the lysosomal lumen. In airway epithelial cells, inhibition of endolysosomal acidification suppresses TLR3-mediated inflammatory signaling (52), but that finding does not by itself demonstrate inhibition of viral entry. Any therapeutic intervention should be guided by the identified cellular compartment and functional defect, while preserving lysosomal degradation and host defense.

Cathepsins and other lysosomal proteases are essential for normal degradation and immune functions in the lung. Although excessive extracellular or damage-associated protease activity may contribute to tissue injury, broad cathepsin inhibition cannot be assumed to be beneficial during lung development because these enzymes also participate in host defense and intracellular turnover. Lung-specific studies are required before protease modulation can be considered a developmental therapeutic strategy.

### 4.3 Developmental safety and clinical translation

Lysosome-targeted delivery may provide a means to improve cellular specificity and reduce systemic exposure. Experimental nanocarriers can exploit endolysosomal trafficking, luminal pH, or intracellular protease activity to deliver cargo to lysosome-associated compartments. For pediatric pulmonary applications, however, the relevant questions extend beyond delivery efficiency: investigators must establish where the material accumulates, how long it persists, which lung cell types internalize it, whether it reaches conventional lysosomes or LROs, and whether it alters surfactant or macrophage function.

Premature lungs provide a particularly demanding translational environment because epithelial, vascular, immune, and surfactant systems are simultaneously undergoing maturation. Lysosomal storage disorders demonstrate that severe lysosomal dysfunction can produce respiratory phenotypes, but these disorders are not direct models of BPD (53).

Future studies should prioritize dynamic autophagic-flux assays, compartment-specific pH measurements, organelle-targeted functional readouts, and causal rescue experiments. Candidate interventions should be tested across developmental time points and evaluated for effects on alveolarization, pulmonary vascular growth, surfactant secretion, epithelial differentiation, immune maturation, and host defense. Noninvasive biomarkers of lysosomal function are also needed, but their validity in neonates remains to be established.

The translational framework can therefore be expressed as a sequence: define the cell and organelle compartment; identify the functional lesion; demonstrate causality; restore the defective process; and then establish developmental safety. This approach is more likely to yield clinically meaningful lysosome-directed strategies than nonspecific manipulation of autophagy or lysosomal activity.

## 5 Conclusions and future perspectives

Lysosomes occupy a central position at the interface of degradation, metabolism, organelle quality control, and inflammatory signaling in the developing lung. Direct pulmonary evidence supports epithelial autophagy in lung morphogenesis (7), AT-II cell autophagy-related pathways in lamellar-body maturation and surfactant homeostasis (8, 9), and the effects of experimentally altered lysosomal lipid handling on surfactant and alveolar structure (10). Disease-model studies identify context-dependent remodeling of autophagic or endolysosomal pathways in hyperoxic injury, BPD, and RSV infection (11, 19, 35, 36, 39, 40). These injury-related mechanisms should not be assumed to direct normal lung development.

At the same time, the current evidence does not justify treating lysosomal dysfunction as a single uniform mechanism. The consequences of pathway perturbation depend on developmental stage, cell type, cargo, compartment, and the balance between lysosomal delivery and degradation. Static LC3-II or p62 measurements are insufficient to define flux, while pH measurements must specify the intracellular compartment. These methodological principles are essential for interpreting both experimental studies and future therapeutic trials.

The most promising direction is therefore a developmentally informed, compartment-specific model of lysosomal biology. In this framework, lysosomes and lysosome-related organelles integrate metabolic and stress signals to maintain AT-II cell competence, surfactant homeostasis, mitochondrial quality, and immune function. When this adaptive network is disrupted by prematurity, hyperoxia, infection, or other stressors, impaired clearance, lipid imbalance, mitochondrial injury, and inflammatory amplification may contribute to defective alveolar and epithelial maturation. This model generates testable hypotheses without assuming that every pediatric lung phenotype is caused by a primary lysosomal defect.

Future work should move from descriptive lysosomal phenotyping toward causal experiments that define the affected compartment and restore its function. Developmental timing, cell-specific delivery, dynamic flux measurements, organelle-resolved lipid and pH assays, and clinically relevant lung models will be particularly important. If these requirements are met, lysosome-centered biology may provide a tractable framework for understanding and eventually treating selected forms of pediatric lung disease.

## Data Availability

The original contributions presented in the study are included in the article/Supplementary Material, further inquiries can be directed to the corresponding author.
